# Effects of Oil Palm Shell Coarse Aggregate Species on High Strength Lightweight Concrete

**DOI:** 10.1155/2014/387647

**Published:** 2014-05-29

**Authors:** Ming Kun Yew, Hilmi Bin Mahmud, Bee Chin Ang, Ming Chian Yew

**Affiliations:** ^1^Department of Civil Engineering, Faculty of Engineering, University of Malaya, 50603 Kuala Lumpur, Malaysia; ^2^Center of Advanced Materials, Department of Mechanical Engineering, Faculty of Engineering, University of Malaya, 50603 Kuala Lumpur, Malaysia

## Abstract

The objective of this study was to investigate the effects of different species of oil palm shell (OPS) coarse aggregates on the properties of high strength lightweight concrete (HSLWC). Original and crushed OPS coarse aggregates of different species and age categories were investigated in this study. The research focused on two OPS species (*dura* and *tenera*), in which the coarse aggregates were taken from oil palm trees of the following age categories (3–5, 6–9, and 10–15 years old). The results showed that the workability and dry density of the oil palm shell concrete (OPSC) increase with an increase in age category of OPS species. The compressive strength of specimen CD3 increases significantly compared to specimen CT3 by 21.8%. The maximum achievable 28-day and 90-day compressive strength is 54 and 56 MPa, respectively, which is within the range for 10–15-year-old crushed * dura* OPS. The water absorption was determined to be within the range for good concrete for the different species of OPSC. In addition, the ultrasonic pulse velocity (UPV) results showed that the OPS HSLWC attain good condition at the age of 3 days.

## 1. Introduction

The environmental impact of oil palm cultivation is a highly controversial topic. Oil palm shells (OPS) are agricultural solid end products of oil palm manufacturing processes. Palm trees grow in regions where the temperature is hot with copious rainfall such as Malaysia, Indonesia, and Nigeria. In Malaysia, oil palm fruits can be classified as* dura*,* tenera,* and* pisifera*.* dura* is a homozygous dominant with thick shells while* pisifera* is a homozygous recessive without shells. A ring of fibres called “mesocarp” surrounds the kernel.* dura* is cross-pollinated with* pisifera* to produce heterozygous* tenera* with an intermediate shell thickness surrounded by a ring of fibres in the mesocarp. It has been shown that high strength lightweight aggregate concrete (HSLWAC) has a compressive strength of typically between 34 and 69 MPa through the incorporation of various pozzolans and water reducers. HSLWAC has a water to cement ratio less than 0.45 and an air-dry density less than 2000 kg/m³ [1]. The utilization of OPS as lightweight aggregate (LWA) in the production of lightweight aggregate concrete (LWAC) has been a topic of research since the early 1984 in Malaysia by Abdullah [2]. Palm oil production is a significant industry in the Malaysian economy, as Malaysia is the world's second largest producer of the commodity in 2012 [3]. The need for vegetable oils is constantly increasing and an increase in the cultivation of palm oil is forecasted in the near future. Malaysia produces 18.79 million tonnes of crude palm oil on roughly 5 million hectares of land [4, 5]. Even though Indonesia produces more palm oil, Malaysia is the world's largest exporter of palm oil, in which 18 million tonnes of palm oil products were exported in 2011. China, Pakistan, the European Union, India, and the United States of America are the primary importers of Malaysian palm oil products [6]. Approximately 80% of the volume from fresh fruit bunch processing is removed as waste [7]. A prevailing problem in the processing of palm oil is the large amounts of by-products produced such as empty fruit bunches (EFB), palm kernel shells (PKS), oil palm shells (OPS), and palm oil mill effluent (POME). These by-products are one of the main contributors to the nation's pollution problem. OPS are one of the wastes produced during palm oil processing. Recently, a large amount of OPS waste materials are stockpiled and dumped, which causes storage problems within the vicinity of factories as large quantities of these wastes are produced every day [8]. In Malaysia, it is estimated that over 4.6 million tonnes of OPS is produced annually as waste [9]. The density of the shells is within the range of a majority of commonplace lightweight aggregates [10] and the specific gravity of the shells ranges between 1.14 and 1.37. A number of studies over the last two decades showed that OPS can be employed as LWA in order to produce structural LWAC, with a reduction in density of 20–25% compared to normal weight concrete [11]. These types of LWA will not only provide significant savings in the overall construction costs, but also address concomitant environmental problems by reducing solid waste. A cost analysis in Nigeria [12] revealed that a cost reduction of 42% is possible for concrete made from OPS. Several studies showed that although the engineering properties of OPSC are generally satisfactory [13–16], there is still reluctance in implementing OPSC compared with other types of LWAC. The reason for this was given by Okafor [10], who concluded that OPS are incapable of producing concrete with a compressive strength above 30 MPa. However, recent studies have shown that high strength LWAC can indeed be produced [17–19].

Most current studies on OPSLWC focused on the investigation of their engineering properties and no information is available regarding the influence of different species and age categories of OPS coarse aggregates on the properties of lightweight concrete. Shafigh et al. [17] reported that the difference between old and fresh OPS is in the fibre content of the surface. The percentage of fibres for old OPS grains is less than 5% compared with fresh OPS grains which usually contain more than 50% fibres. The percentage of fibres increases the demand for water and diminishes the contact area between the mortar and OPS surface, which results in a weak interfacial transition zone. This in turn, affects the bonding strength between the OPS and mortar phase. A new method for producing high strength oil palm shell lightweight concrete was implemented by Shafigh et al. [18]. The flakiness of large-sized OPS aggregates is reduced significantly by crushing these aggregates which improve the performance of coarse aggregates and increases compressive strength. However, it has been shown that the different species and age categories of OPS coarse aggregates have the most significant impact on the production of high strength OPS lightweight concrete. Considerable research has been devoted on the application of various species and age categories of OPS aggregates to enhance the properties of OPS HSLWC. The methods proposed based on the results of such studies are commonly termed “OPS selection methods.” OPS selection method is considered to be an environmental-friendly alternative compared to chemically impregnated OPS materials [20] in enhancing the properties of HSLWC. Hence, this study was motivated based on the foundation that different species of OPS aggregates possess better characteristics which will offer interesting opportunities in the production of OPS HSLWC and forms the objective of this study.

## 2. Materials and Methods

### 2.1. Materials

#### 2.1.1. Cement

The cement used in this study was ASTM type I ordinary portland cement (OPC) [21] with a specific gravity of 3.14 g/cm³ and a Blaine's specific surface area of 3510 cm²/g. The cement content was kept constant at 550 kg/m^3^. Silica fume (SF) having 5% cement by weight and a specific gravity of 2.10 g/cm³ was used as the supplementary cementitious material. To enhance the mechanical properties of the concrete, densified SF was added as additional mineral admixture. The silica fume used for this research investigation was procured from Scancem Materials Sdn. Bhd. The chemical composition and physical properties of OPC are presented in Table 1.

#### 2.1.2. Water and Superplasticizer (SP)

Potable water with a pH value of 6 was used for all mixes. Polycarboxylic ether (PCE) supplied by BASF was used as the superplasticizer (SP) in this study complies with the ASTM: C494/C494M-13 standard. The SP was mixed at a constant amount of 1.0% of the cement weight in order to facilitate workability. A water-to-binder ratio of 0.3 was used for all mixes.

#### 2.1.3. Fine and Coarse Aggregates

Local mining sand was used as the fine aggregate, having a specific gravity, fineness modulus, water absorption, and maximum grain size of 2.68 g/m^3^, 2.72, 0.97%, and 4.75 mm, respectively. The fine aggregate content was kept constant at 860 kg/m^3^ for all mixes.

**Figure 1:**
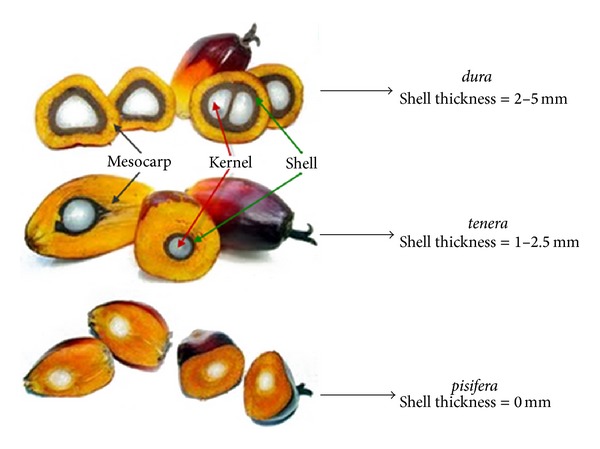
Species of OPS coarse aggregates.

**Figure 2:**
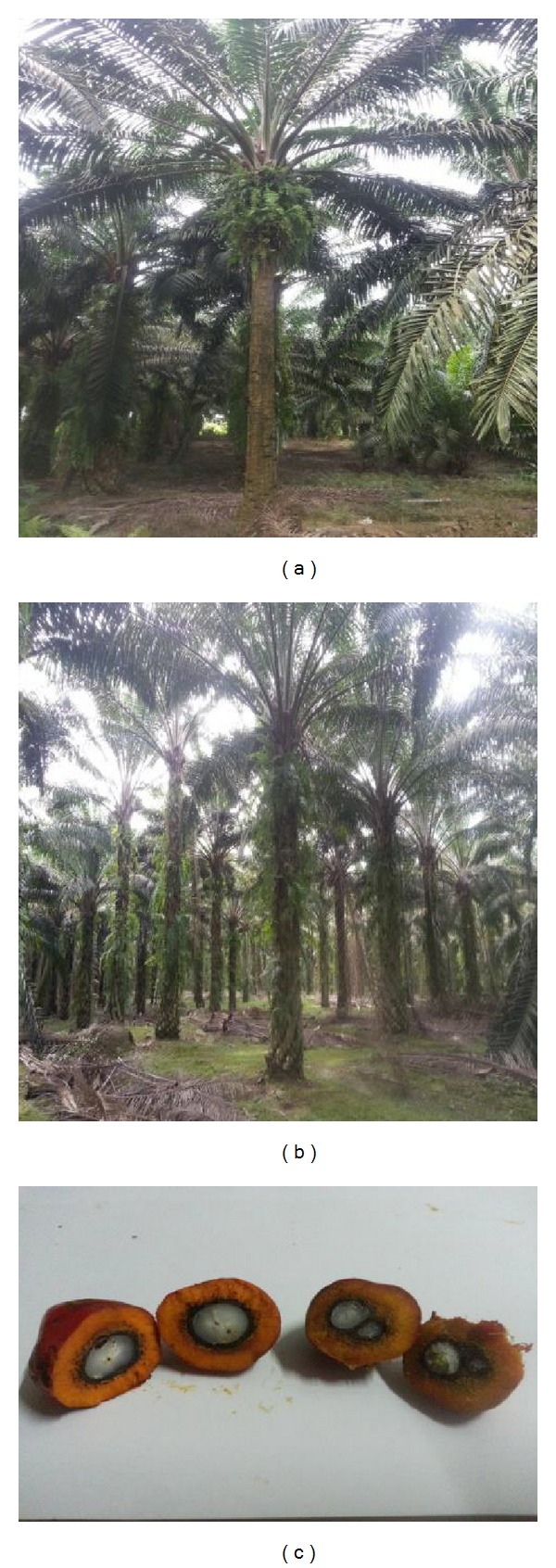
*dura* (a) and* tenera* (b) OPS aggregates taken from 10–15-year-old oil palm trees.

**Figure 3:**
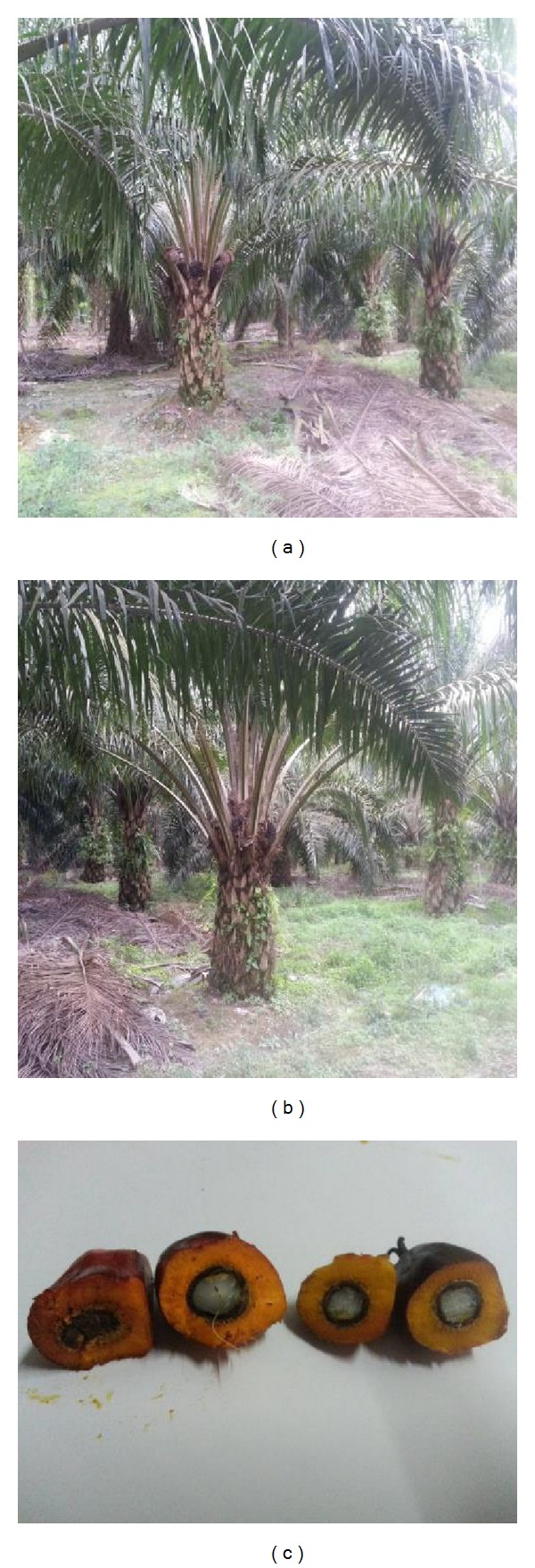
*dura* (a) and* tenera *(b) OPS aggregates taken from 6–9-year-old oil palm trees.

**Figure 4:**
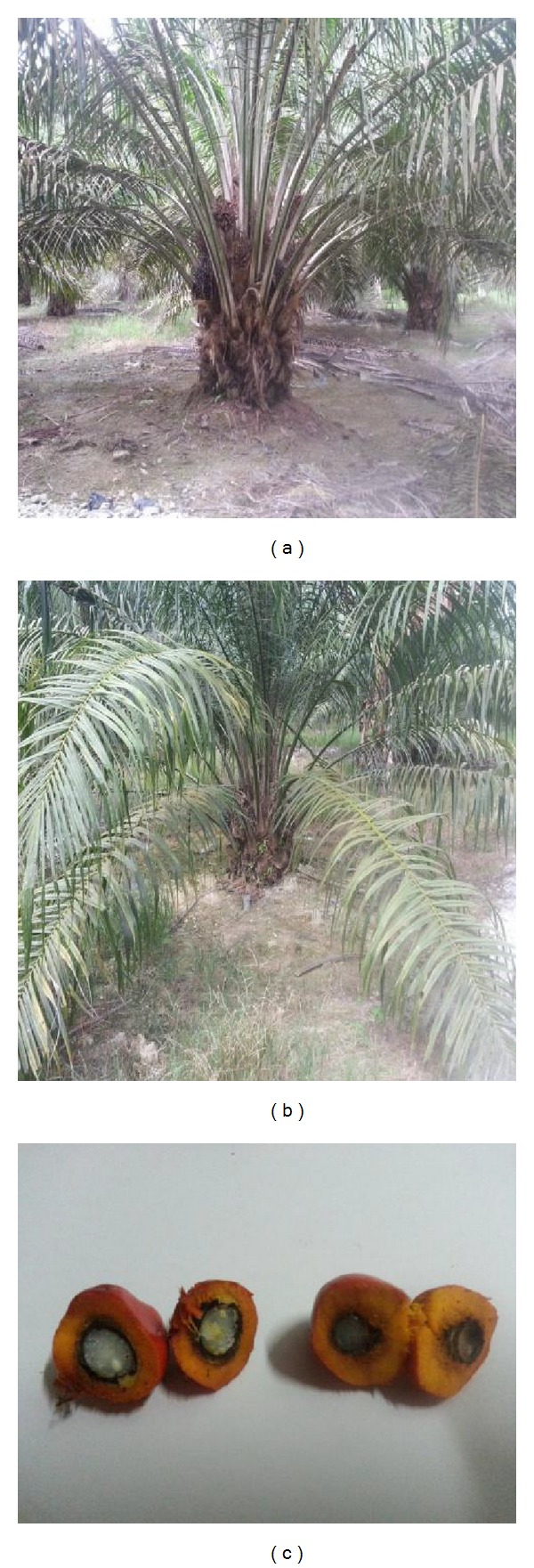
*dura* (a) and* tenera* (b) OPS aggregates taken from 3–5-year-old oil palm trees.

**Figure 5:**
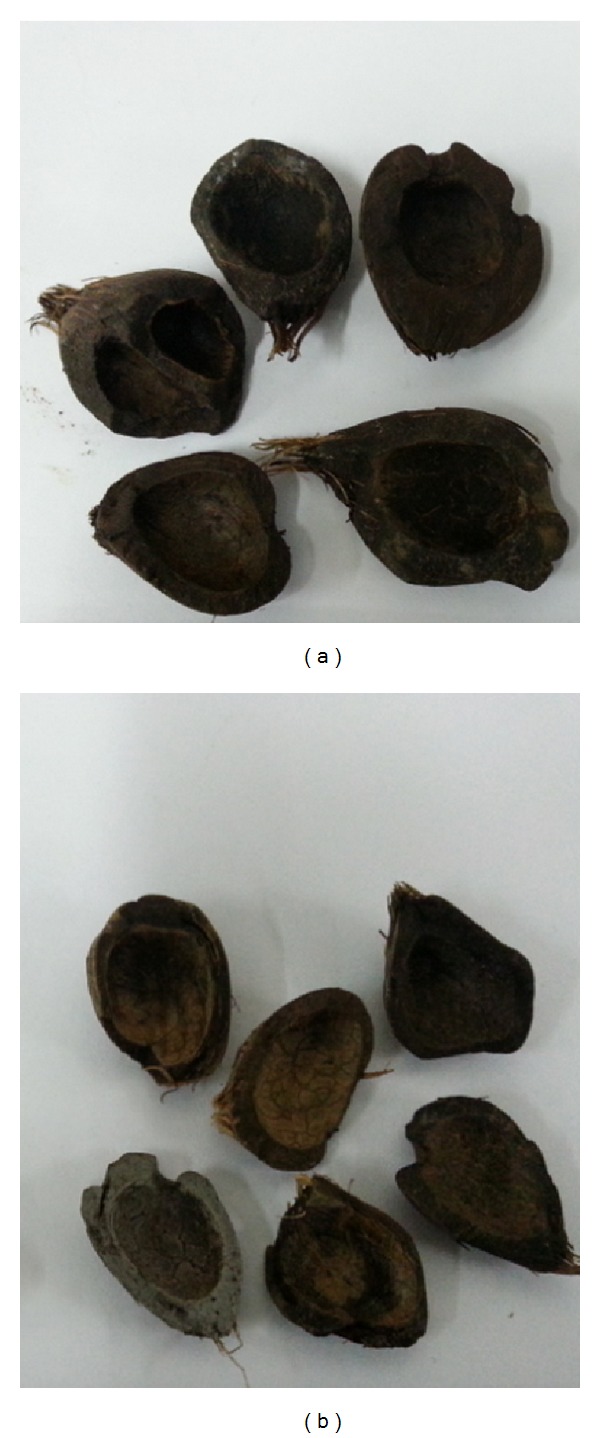
Original* dura* (a) and* tenera* (b) OPS coarse aggregates.

**Figure 6:**
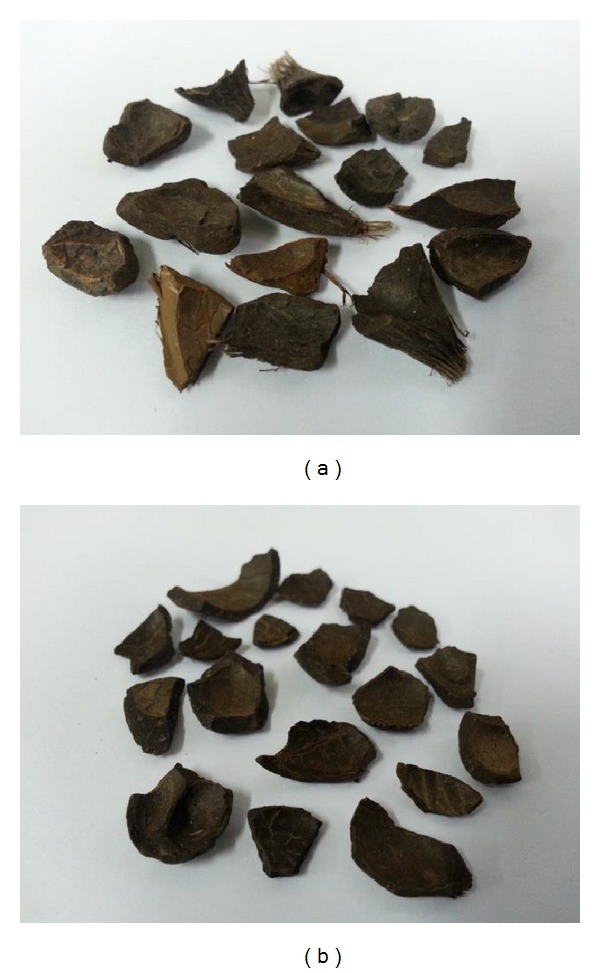
Crushed* dura* (a) and* tenera *(b) OPS coarse aggregates.

OPS were used as the coarse aggregate in this study. Different species of* dura*,* tenera *and* pisifera* OPS are shown in Figure 1, while different age categories of* dura* and* tenera* used are shown in Figures 2, 3, and 4. The OPS aggregates used in this study are classified as “immature” from 3 to 5 years of age, “young mature” from 6 to 9 years of age, and “young prime” from 10 to 15 years of age. The OPS were collected from a local crude palm oil factory in both original and crushed conditions, as shown in Figures 5 and 6, respectively. It can be seen that the original OPS have concave and convex shapes with a smooth surface on the outer convex side, whereas the crushed OPS have more spiky edges compared to the original OPS. The OPS aggregates were subsequently air dried in the laboratory to attain an approximately saturated surface dry condition. The aggregates were sieved using a 12.5 and 9.5 mm sieve in order to investigate the effect of OPS aggregate size on the compressive strength of the concrete. This yields OPS aggregates with a different maximum size. A comparison between the physical properties of OPS aggregates and crushed granite is presented in Table 2, while the grading of OPS aggregates is presented in Table 3. The OPS content was kept constant at 380 kg/m^3^ for all mixes.

### 2.2. Mix Proportions

A total of 12 mixes were prepared in this study and the mix proportions are presented in Table 4. The dosage of water and SP was kept constant for all mixes.

### 2.3. Testing Methods and Curing Regimes

The procedure used to prepare OPSC is detailed as follows. Firstly, the sand and OPS were poured into a concrete mixer and dry-mixed for 1 min. Secondly, the cement was spread and dry-mixed for 1 min. Water and superplasticizer were then added and mixed for 5 min. Slump test was performed on the mixture prior to sample casting. The fresh concrete mix was placed into 100 mm cube steel oiled moulds and a poker vibrator was used to eliminate air bubbles in the mixture. The specimens were demoulded approximately 24 hours after casting and were cured in water at 25 ± 2°C until the age of testing. The compressive strength was determined at 1, 3, 7, 14, 28, 56, and 90 days using a compression testing machine with a capacity of 3000 kN and equipped with a rate of loading controller, in accordance to the British Standard (BS) 1881. Three concrete cubes were cast for each mixture in order to determine the average value at a particular age. In order to determine the water absorption of all mixes at the age of 28 days, the specimens were dried in an oven at 100 ± 5°C until a constant mass was reached and then fully immersed in water at 22 ± 2°C for 72 h. This test is similar to the test conducted by Razak et al. [24], Teo et al. [25], and Shafigh et al. [17].

The specimens were cured under seven types of curing condition to determine the effect of curing environment on the 28-day compressive strength of high strength OPSC. A brief description of each curing condition is given as follows:

- WC: the specimens were immersed in water at 22 ± 2°C after demoulding until the age of testing;
- 14W: the specimens were cured in water for 13 days after demoulding and then air-cured in laboratory environment with a relative humidity of 60 ± 10% and temperature of 29 ± 3°C;
- 2T14D: the specimens were watered twice per day (once in the morning and afternoon) for 13 days after demoulding and then air-cured in laboratory environment;
- 7W: the specimens were cured in water for 6 days after demoulding and then air-cured in laboratory environment with a relative humidity of 60 ± 10% and temperature of 29 ± 3°C;
- 2T7D: the specimens were watered twice per day (once in the morning and afternoon) for 6 days after demoulding and then air-cured in laboratory environment;
- 2T3D: the specimens were watered twice per day (once in the morning and afternoon) for 2 days after demoulding and then air-cured in laboratory environment;
- AC: the specimens were stored in laboratory environment after demoulding.

## 3. Results and Discussion

### 3.1. Properties of Fresh Concrete (Workability)

**Figure 7:**
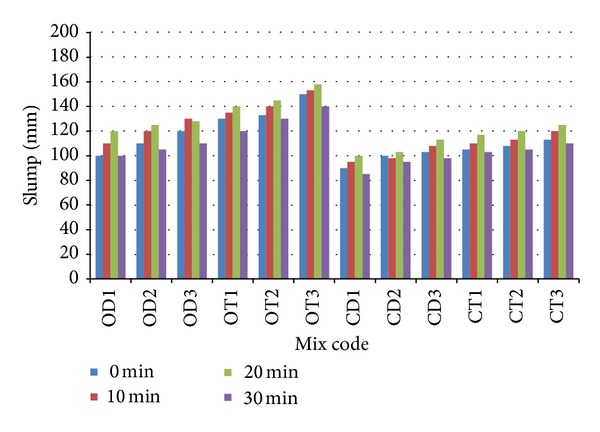
Effect of OPS species (original and crushed) and age categories on workability.

Slump test was carried out to determine the consistency of fresh concrete. The effect of original and crushed OPS species and age category on the slump values of fresh concrete specimens is summarized in Figure 7. The specimens were tested immediately after mixing as well as at 10, 20, and 30 min to determine the slump loss of the mixtures. It can be observed that the concrete mixes achieved maximum slump at 20 min, which indicates that a longer period is required to enable the SP to react with fresh concrete in order to achieve good workability for OPSC. It was found that the workability of fresh OPSC improves with an increase in age category and varies from crushed OPS to original OPS aggregates.

In general, all mixes exhibit “good” to “high workability” within a range of 90–150 mm. It can be seen that all the concretes mixes had a maximum slump at 10 min or 20 min. This phenomenon shows that to achieve better workability for OPSC it needs more time for mixing. The OT3 mix had the highest slump value of 158 mm at 20 min. Segregation and bleeding were not observed in these mixes, as it is possible that the highest age category for the original* tenera* OPS aggregates reduces water absorption, which increases the workability and flow of the concrete. The CD1 mix had the lowest slump value among the mixes, because the maximum size of the crushed OPS aggregates of the mix was reduced from 12.5 to 9.5 mm. This affects the workability of the mix because it contains variety of irregular shapes of the OPS which prevented full compaction from being achieved, as well as the higher water absorption of OPS. Furthermore, it can be observed that, from the time of leaving the mixer, all the concrete mix exhibited slump loss at 30 min. A previous study reported that the concrete slump increases slightly when the maximum size of the OPS aggregates increases from 9.5 to 12.5 mm [17]. The strength of the interfacial transition zone tends to increase by improving the workability and compatibility of the mixture, which increases the strength of the concrete.

### 3.2. Density

**Figure 8:**
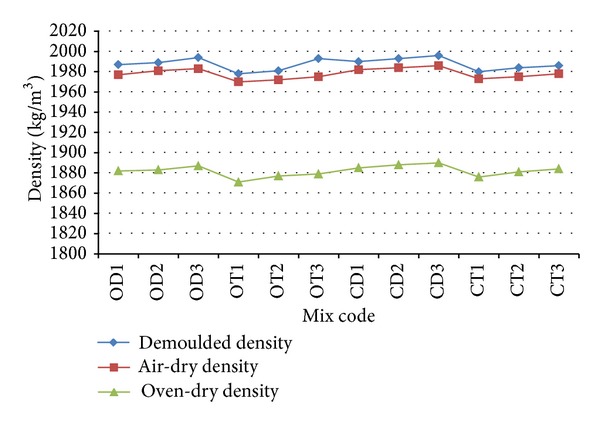
Variations of demoulded density, air-dry density, and oven-dry density for concrete mixes made from different species of OPS (original and crushed) and age categories.

Three types of density (demoulded density, 28-day air-dry density, and oven-dry density) were measured for all mixes. Structural lightweight concrete (LWC) is typically defined as concrete with an oven-dry density less than 2000 kg/m³ [23]. It was found that the density of all the mixes is within the range of structural LWC for all mixes, as shown in Figure 8. The 28-day air-dry density and oven-dry density for the original and crushed OPS mixes range between 1871–1876 kg/m³ and 1983–1986 kg/m³, respectively. Assuming that the density for normal weight concrete (NWC) is 2350 kg/m^3^, the 28-day air-dry density and oven-dry density for all mixes are approximately 20% and 16% lower than that of ordinary concrete, respectively.

An increase in age category for the original* dura* OPS species from 3–5 years old to 6–9 and 10–15 years old slightly increases the demoulded density, air-dry density, and oven-dry density by 0.2% and 0.3% at 1 d, 0.1% and 0.2% at 3 d, or 7 d and 0.2% and 0.3% at 28 d, respectively. A similar trend can be observed for other OPSC mixes, as shown in Figure 8.

### 3.3. Compressive Strength

#### 3.3.1. Continuous Moist Curing

The results of the compressive strength for all concrete mixes subjected to moist curing up to 90 days are presented in Table 5. It was found that the CD3 concrete mix had the highest compressive strength, with a value of 54 and 56 MPa at 28 d and 90d, respectively. The compressive strength increases with an increase in age category for different species of original and crushed OPS at all ages. The mixes of CD1 to CD3 increase the compressive strength by 4.8% and 8.0% at 1 d, 7.5% and 11.3% at 3 d, 5.6% and 11.3% at 7 d, 5.0% and 9.9% at 14 d, 5.1% and 6.5% at 28 d, and 1.6% and 5.9% at 90 d, respectively. This observation may be attributed to the thicker and larger (and hence harder and stronger) OPS aggregates with respect to the age category of the oil palm trees. The compressive strength increases by 9.0%, 11.6%, and 16.5% from OD1–OD3 to CD1–CD3 at 28 d, when the maximum size of OPS aggregates changes from original (12.5 mm) to crushed (9.5 mm). From the results, it is evident that the flakiness index of the aggregates reduces significantly and a higher stiffness is attained when the OPS aggregates were crushed to smaller sizes, particularly for the CD3 mix. A previous study reported that the transition zone will weaken significantly when large aggregates are used, due to internal bleeding and development of microcracks, which results in lower compressive strength [26]. Although the surface texture of the concave and convex faces of* dura* and* tener*a OPS is fairly smooth, the broken edges of* dura* OPS are rough and spiky compared to* tenera* OPS, which strengthens the physical bond between the aggregates and hydrated cement paste.

From Table 5, it can be observed that all mixes achieved high early strength at 7 days of age. The mixes reached 64–69%, 32–46%, and 82–89% of the 28-day compressive strength at 1 d, 3 d, and 7 d, respectively. The high early strength may be attributed to the addition of silica fume since the pozzolanic reaction and microfiller effects of the silica fume contribute to the early strength of OPSC. The addition of silica fume improves bonding within the OPSC and helps reduce permeability. The silica fume reacts with the weak leachable calcium hydroxide (CaOH) liberated from hydration of the cement to form strong dense calcium silicate hydrate (C–S–H). As a microfiller, the extreme fineness of the silica fume allows it to fill the microscopic voids between the cement particles. This greatly reduces permeability and improves the OPS aggregate-cement paste in the interfacial zone, and, consequently minimizes the induction of microcracks compared to conventional concrete.

An additional benefit of OPS aggregate selection can be observed from the compressive strength of the CD3 mix, whereby the cement content of this mix is 550 kg/m³, which gives a 28-day compressive strength of 54 MPa. Alengaram et al. [27] produced grade 30 OPSC, in which cement having a density range of 504–564 kg/m³ was mixed with 5% fly ash and 10% silica fume as additional cementitious materials. The total cementitious material of their study was within the range of 585–654 kg/m³. It is evident that the cement content of the CD3 mix falls within the range used by Alengaram et al. [27] and that the CD3 mix contains 5% supplementary cementitious materials that are lower compared to their study. This indicates that the methodology adopted in this study yields a compressive strength that is significantly higher for OPSC than that obtained by Alengaram et al. [27].

### 3.4. Effect of Curing Condition on the 28-Day Compressive Strength

**Figure 9:**
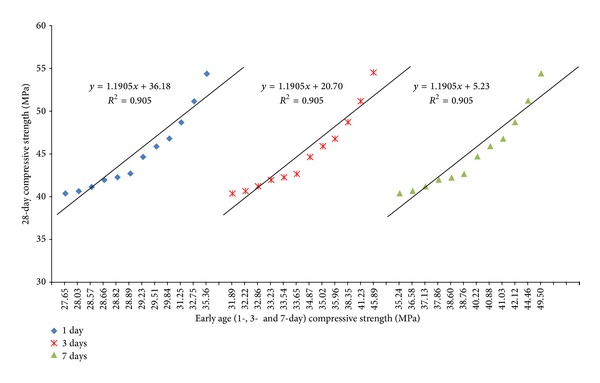
Effect of curing conditions on the 28-day compressive strength of concrete mixes.

Curing can be defined as a procedure used to ensure the hydration of Portland cement in newly placed concrete. In general, curing implies the control of moisture loss and effects of temperature in concrete during its early stages so that the desired properties may develop [28]. As a general rule, at least 14 days of good curing is necessary for OPSC. Although a minimum period of 7 days of moist curing is generally recommended for concrete containing normal Portland cement or a mineral admixture, a longer curing period is desirable to ensure higher strength contribution from pozzolanic reactions [29]. The 28-day compressive strength of specimens subjected to seven curing conditions is shown in Figure 9. It can be observed that all concrete mixes experienced a strength loss in air curing or early curing regimes compared to those that were water-cured continuously, regardless of mix proportions. The compressive strength loss for the 28-day specimens under air curing (AC) is within the range of 12.7–17.9%, while the values are within 5.3–16.0% under partial early curing (2T3D, 2T7D, and 2T14D). The compressive strength loss for the 28-day specimens is within 8.9–13.6% and 4.9–8.2% under early curing of 7W and 14W, respectively. The average 28-day compressive strength under AC, 2T3D, 2T7D, 7W, and 14W curing conditions is approximately 84%, 87%, 88%, 89%, 93%, and 94% of that for concrete under full water curing condition, respectively. The high early strength of the OPSC may also be contributed by the highly reactive and microfiller effects of the silica fume.

**Figure 10:**
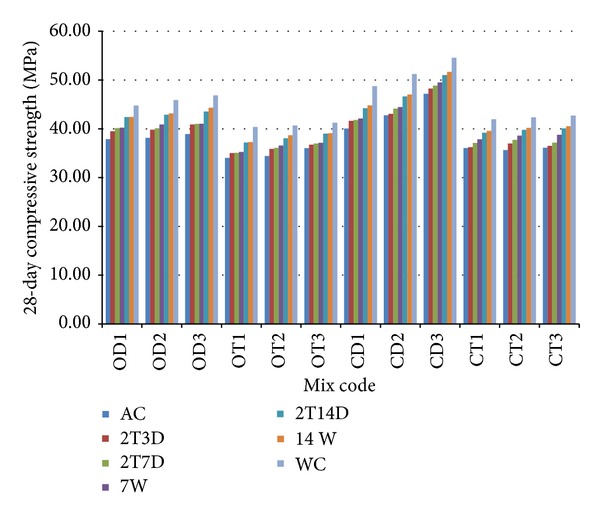
Relationship between early age (1-, 3-, and 7-day) and 28-day compressive strength for mixes made from different OPS species (original and crushed) and age categories.

The relationship between the early-age strength and 28-day strength for different species of OPSC and age categories is presented in Figure 10. It is observed that there is an acceptable linear relationship between the early age and 28-day strength for mixes with different OPS species and age categories for crushed and original OPS aggregates, with a high correlation coefficient. Such a high correlation coefficient could also be observed for OPSC made with crushed OPS aggregates [24]. Furthermore, high correlation coefficients were also observed for OPSC made from OPS aggregates subject to heat treatment [19].

### 3.5. Water Absorption

**Figure 11:**
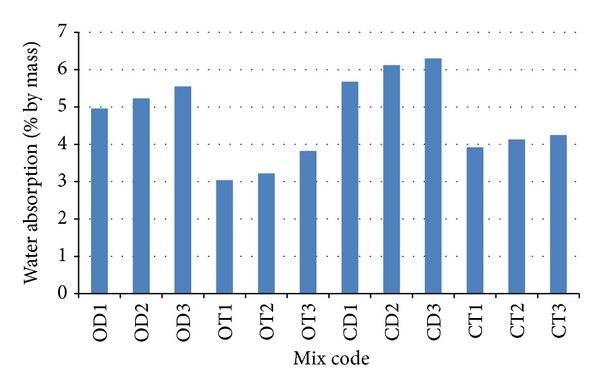
Water absorption of various concrete mixes made from different OPS species and age categories.

The water absorption for all mixes is given in Figure 11. In this study, it was observed that the lowest water absorption value is 3.04% while the highest water absorption value is 6.30% for high strength OPSC. Moreover, it can be seen that the water absorption for crushed OPS species is higher with an increase in age category compared to the original OPS species. This may be due to the broken edges of the crushed OPS species that are rough and spiky, and therefore the crushed aggregates tend to absorb water. In contrast, the surface texture of the concave and convex faces of the original OPS species is fairly smooth, which may reduce water absorption. It has been stated that most good concrete have water absorption below 10% by weight [30]. Thus, it can be deduced that the water absorption value measured for the OPSC in this study falls within the range of good concrete. It has been reported that the water absorption for expanded polystyrene aggregate concrete [31] and pumice aggregate concrete [32] is within the range of 3–6% and 14–22%, respectively.

### 3.6. Ultrasonic Pulse Velocity (UPV)

**Figure 12:**
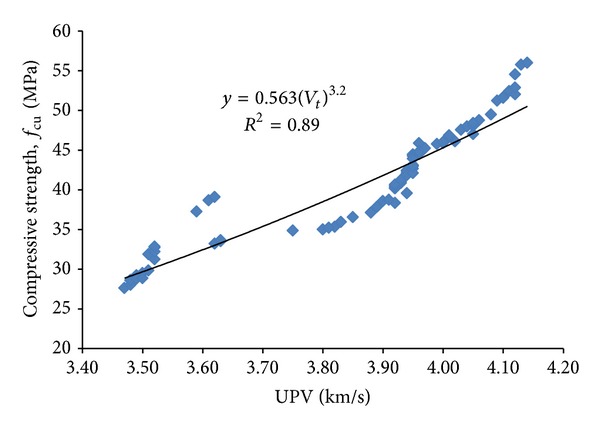
Relationship between compressive strength and ultrasonic pulse velocity.

UPV is a nondestructive method used to evaluate the quality of concrete. This method can be used to detect internal cracking and other defects, as well as changes in concrete such as deterioration due to aggressive chemical environment, freezing, and thawing. This method makes it possible to estimate the strength of concrete test specimens and in-place concrete. Based on the UPV values in Table 6, it is evident that the specimens of different OPS species and age categories have an insignificant effect on the UPV values of OPS LWHSC. However, it was found that UPV can be correlated with its corresponding compressive strength, as shown in Figure 12, with a *R* ^2^ value of 0.89. Equation (1) is proposed to estimate the compressive strength of OPSC based on the UPV values

(1) $$\begin{array}{c} f_{\text{cu}}=0.563{\left(V_{t}\right)}^{3.2}, \end{array}$$

where *f*_cu_ represents the cube compressive strength (MPa) and *V*_*t*_ represents the transverse ultrasonic pulse velocity (km/s).

## 4. Conclusions

The following conclusions are derived based on the results presented in this paper.

1. A high early compressive strength was achieved for all mixes in this study, which may be due to the addition of silica fume. The addition of silica fume greatly reduces permeability and improves aggregate-cement paste interface of the concrete and consequently minimizes the induction of microcracks.
2. The workability of concrete can be enhanced by increasing the age category of OPS aggregates. In general, all mixes achieved good and high workability.
3. Increasing the age category of OPS aggregates slightly increases the density of the concrete. The average 28-day air-dry density and oven-dry density for the different species of OPSC is within 1970–1986 kg/m³ and 1871–1890 kg/m³, respectively.
4. It is possible to produce OPS HSLWC with a 28-day compressive strength of 55 MPa by selecting crushed* dura* OPS aggregates taken from oil palm trees aged between 10 and 15 years old.
5. OPS aggregates can be used as an environmental-friendly alternative to enhance the compressive strength of OPSLWC, provided that suitable OPS species and age category as well as size of the OPS aggregates are selected.
6. There is a linear relationship with high correlation coefficient between the early age and 28-day compressive strength for OSPC made from aggregates of different species and age category.
7. All OPS HSLWC are sensitive to poor curing. A period of 14 days of partial water curing is recommended for this type of LWAC. The period for partial water curing compressive strength appears to be very similar to that for continuous curing.
8. The water absorption value varies from 3.04 to 6.40% for all OPSC mixes, which falls within the range of good concrete.
9. Based on the UPV test results, concrete mixes made from different OPS species and age categories can be categorized as attaining good condition after 3 days.
10. OPS lightweight aggregates showed the possibility and acceptable performance for potential application in concrete structures. The substitution of OPS as full replacement for conventional aggregate contributes significantly to green building rating systems, a sustainable infrastructure, and mitigating of the environmental impact of the construction industry.

## Figures and Tables

**Table 1:** Chemical composition and physical properties of OPC.

Chemical composition (%)						Physical properties		
SiO_2_	Fe_2_O_3_	CaO	MgO	Al_2_O_3_	SO_3_	LOI	Specific gravity	Blain specific surface area (cm²/g)
21.28	3.36	64.64	2.06	5.6	2.14	0.64	3.14	3510

**Table 2:** Comparison between the physical properties of OPS aggregates and crushed granite [22].

Physical property	Original *dura* OPS	Original* tenera *OPS	Crushed *dura* OPS	Crushed *tenera* OPS	Crushed granite [23]
Maximum size (mm)	12.5	12.5	9.5	9.5	—
Specific gravity (saturated surface dry)	1.28–1.33	1.15–1.22	1.30–1.37	1.19–1.27	2.61
Compacted bulk density (kg/m³)	612–670	596–617	620–684	608–624	1470
Water absorption (24 h) (%)	24.8	18.9	19.6	17.9	0.76
Aggregate impact value (%)	2.10	2.03	2.60	2.35	17.29

**Table 3:** Grading of OPS aggregates.

Sieve size (mm)	Cumulative % by weight passing sieve size			
	Original *dura* OPS (12.5 mm)	Original *tenera *OPS (12.5 mm)	Crushed *dura* OPS (9.5 mm)	Crushed *tenera* OPS (9.5 mm)
20	100	100	100	100
12.5	100	100	100	100
9.5	82.28	83.22	100	100
8	58.26	59.27	93.52	94.62
4.75	23.45	22.78	24.13	23.65
2.36	4.41	4.28	5.39	5.34

**Table 4:** Different species of OPS and the age categories of original and crushed OPS mixes.

Number	Mix code	OPS species	Age categories (years old)
Original OPS			
1	OD1	*dura *	3 to 5
2	OD2	*dura *	6 to 9
3	OD3	*dura *	10 to 15
4	OT1	*tenera *	3 to 5
5	OT2	*tenera *	6 to 9
6	OT3	*tenera *	10 to 15
Crushed OPS			
7	CD1	*dura *	3 to 5
8	CD2	*dura *	6 to 9
9	CD3	*dura *	10 to 15
10	CT1	*tenera *	3 to 5
11	CT2	*tenera *	6 to 9
12	CT3	*tenera *	10 to 15

**Table 5:** Development of compressive strength of lightweight OPSC under continuous moist curing.

Mix code	Compressive strength (MPa)^a^						
	1 d	3 d	7 d	14 d	28 d	56 d	90 d
OD1	29.2 (65.32)	34.9 (77.92)	40.2 (87.21)	42.5 (94.86)	44.8	46.1 (103.06)	48.0 (107.24)
OD2	29.5 (64.29)	35.0 (76.30)	40.9 (87.63)	43.1 (93.97)	45.9	46.7 (101.63)	48.1 (104.66)
OD3	29.8 (63.73)	36.0 (76.80)	41.0 (86.27)	44.3 (94.57)	46.9	47.6 (101.58)	48.4 (103.40)
OT1	27.7 (68.49)	31.9 (78.99)	35.2 (84.21)	37.3 (92.32)	40.4	41.9 (103.67)	43.0 (106.59)
OT2	28.0 (68.92)	32.2 (79.22)	36.6 (86.83)	38.7 (95.11)	40.7	42.1 (103.59)	44.8 (110.08)
OT3	28.6 (69.24)	32.9 (79.64)	37.1 (86.39)	39.1 (94.76)	41.3	43.0 (104.17)	45.2 (109.65)
CD1	31.3 (64.09)	38.4 (78.65)	42.1 (81.66)	44.8 (91.84)	48.8	51.6 (105.78)	52.0 (106.73)
CD2	32.8 (63.93)	41.2 (80.48)	44.5 (84.78)	47.0 (91.78)	51.2	52.4 (102.36)	52.9 (103.24)
CD3	35.4 (64.82)	45.9 (84.12)	49.5 (88.74)	51.7 (94.70)	54.4	55.8 (102.25)	56.0 (102.69)
CT1	28.7 (68.29)	33.2 (79.18)	37.9 (87.99)	39.6 (94.28)	42.0	43.0 (102.53)	45.8 (109.08)
CT2	28.8 (68.05)	33.5 (79.20)	38.6 (87.87)	40.2 (94.92)	42.4	43.9 (103.73)	46.0 (108.64)
CT3	28.9 (67.69)	33.7 (78.84)	38.8 (87.06)	40.5 (95.00)	42.7	44.5 (104.31)	46.9 (109.82)

^a^The data in parentheses are percentage of 28 d compressive strength.

**Table 6:** Ultrasonic pulse velocity (UPV) for concrete mixes made from different OPS species and age categories.

Mix code	UPV (km/s)						
	1 d	3 d	7 d	14 d	28 d	56 d	90 d
OD1	3.49	3.75	3.92	3.94	3.96	4.02	4.04
OD2	3.50	3.80	3.93	3.95	4.00	4.01	4.05
OD3	3.51	3.83	3.93	3.95	4.01	4.03	4.05
OT1	3.47	3.51	3.81	3.59	3.92	3.94	3.95
OT2	3.48	3.52	3.85	3.61	3.92	3.95	3.96
OT3	3.48	3.52	3.88	3.62	3.93	3.95	3.97
CD1	3.52	3.92	3.94	3.96	4.06	4.10	4.12
CD2	3.52	3.93	3.95	4.05	4.09	4.11	4.12
CD3	3.82	3.96	4.08	4.10	4.12	4.13	4.14
CT1	3.48	3.62	3.89	3.94	3.94	3.95	3.99
CT2	3.49	3.63	3.90	3.92	3.94	3.95	4.00
CT3	3.50	3.63	3.91	3.92	3.95	3.96	4.01
